# Superhydrophobic Coating Based on Porous Aluminum Oxide Modified by Polydimethylsiloxane (PDMS)

**DOI:** 10.3390/ma15031042

**Published:** 2022-01-28

**Authors:** Klaudia Olkowicz, Zofia Buczko, Barbara Nasiłowska, Kamil Kowalczyk, Joanna Czwartos

**Affiliations:** 1 Air Force Institute of Technology, 01-494 Warsaw, Poland; kamil.kowalczyk@itwl.pl; 2 Łukasiewicz—Institute of Precision Mechanics, 01-796 Warsaw, Poland; zofia.buczko@imp.lukasiewicz.gov.pl; 3 Institute of Optoelectronics, Military University of Technology, 00-908 Warsaw, Poland; barbara.nasilowska@wat.edu.pl (B.N.); joanna.czwartos@wat.edu.pl (J.C.)

**Keywords:** superhydrophobicity, aluminum, anodization, PDMS, anti-icing, anti-fouling, coating

## Abstract

The aim of this study was to obtain a superhydrophobic coating by modifying anodized aluminum using polydimethylsiloxane (PDMS). In order to obtain a superhydrophobic coating on an aluminum substrate, a multistage treatment was implemented. Specimens of aluminum were treated by abrasive blasting, anodization in sulfuric acid, impregnation by PDMS, rinsing in toluene to remove excess of PDMS, and curing. A rough surface with an additional low free energy layer on it resulted in a superhydrophobic effect. The coating obtained has an average contact angle of 159°. The specimens were tested in terms of durability in natural conditions. Additionally, anti-icing and anti-fouling properties were evaluated. The coating was compared with anodized aluminum obtained by a basic process.

## 1. Introduction

In recent years, surface modifications by superhydrophobic coatings have aroused great interest. A given material becomes water-repellent when the contact angle value of the surface is higher than 150° as a measure of superhydrophobic effect. The contact between the solid surface and the liquid is minimized in that case. The theoretical background concerning wettability and the contact angle (Figures S1 and S2) can be found in the Supplementary Materials.

The superhydrophobic properties of coatings may lead to many applications, such as surfaces with improved corrosion resistance [1]. Another important surface parameter is the sliding effect. A freely sliding drop of water from a water-repellent surface collects dirt from it, which gives the surface self-cleaning properties [2]. This is important from the point of view of architectural applications, for example. As a result of low contact between solids and water, heat exchange is obscured. Therefore, ice formation is delayed, and these surfaces have potential anti-icing properties [3]. In the case of aircraft and wind turbines, effective methods for developing anti-icing surfaces are constantly being sought. Additionally, superhydrophobic surfaces are tested for anti-fouling properties. Superhydrophobic and anti-fouling surfaces have potential applications in environmental sensors [4]. For materials deployed in the marine environment, it is often desirable to meet all the above-mentioned requirements. 

There are many methods of obtaining superhydrophobic surfaces described in the available literature, which may generally be characterized as chemical, electrochemical, and mechanical. Some methods may be very simple, such as obtaining a superhydrophobic surface by blackening using a candle flame [5,6,7] or etching aluminum in lauric acid–ethanol solution [8]. However, as technology evolves, the processes of manufacturing superhydrophobic coatings become more and more advanced. A structure exhibiting superhydrophobic properties may be obtained by microstructuring metal or polymer substrates using laser [9,10,11] or lithography techniques [12,13].

In the study presented by P. Reach et al. [14], it was pointed out that naturally occurring biological surfaces with hydrophobic properties have hierarchical structures. These hierarchical structures are characterized by multiple-scale roughness. A perfect example of a naturally occurring superhydrophobic surface with a hierarchical structure is the lotus leaf. Researchers often try to reproduce the characteristic structure of the lotus leaf on manufactured surfaces [15]. The use of fresh lotus leaves as a template for the production of stamps and then the transfer of this characteristic structure on the substrate [16,17,18,19] is very common. However, other hierarchical structures that do not resemble the structure of a lotus leaf can also exhibit superhydrophobic properties—it is simply important to obtain multiple-scale roughness. For example, multiple-scale roughness may be obtained during the electrodeposition of metal coatings [20,21,22].

Naturally occurring hydrophobic and superhydrophobic surfaces owe their properties not only to their specific structural characteristics but also to the presence of waxes on the surface. Following this, obtaining a superhydrophobic surface is possible by the formation of a suitable structure and its impregnation by a substance with low surface energy. Coatings based on nonporous aluminum oxide obtained during the anodization process are ideal for impregnation. The most popular substances in this regard are fatty acids and polymers [14,23,24,25,26].

The aim of this study was to obtain a superhydrophobic coating on aluminum using three consecutive methods: abrasive blasting (mechanical), anodization of aluminum (electrochemical), and impregnation of porous coating by polydimethylsiloxane (chemical). Polydimethylsiloxane is a polymer with a wide range of applications due to its chemical stability, resistance to biodegradation, optical transparency, and favorable mechanical properties [27]. Many publications describe the use of polydimethylsiloxane (PDMS) to achieve superhydrophobic coatings (including coatings on aluminum), using anodized aluminum oxide as a template to form PDMS nanopillars, and using PDMS in the production of stamps to transfer a lotus leaf structure onto a selected substrate [16,17,18,28,29,30,31,32,33,34,35,36,37]. However, using PDMS to impregnate a porous aluminum coating in order to obtain a superhydrophobic surface in a process similar to the one described within this paper has not been recognized in the available literature. The hydrophobic and superhydrophobic surfaces may be applied as anti-icing surfaces [3]. In the available literature, various types of coatings based on PMDS are characterized by icing delay times of 298 s at −10 °C [38], 600 s at −10 °C [39], 1472 s at −15 °C [40], 1800 s at −5 °C [41], 1380 s at −10 °C [41], 210 s at −15 °C [41], and 673 s from room temperature to −10 °C [42]. Polydimethylsiloxane is also used to create anti-fouling surfaces [31,43,44,45]. Therefore, the coatings obtained have also been evaluated in terms of anti-fouling properties. 

In this study, the variation of contact angle and surface tension of a superhydrophobic coating was evaluated in a durability test in natural conditions and in an anti-fouling test, which is not often found in the available literature.

## 2. Materials and Methods

**Figure 1:**
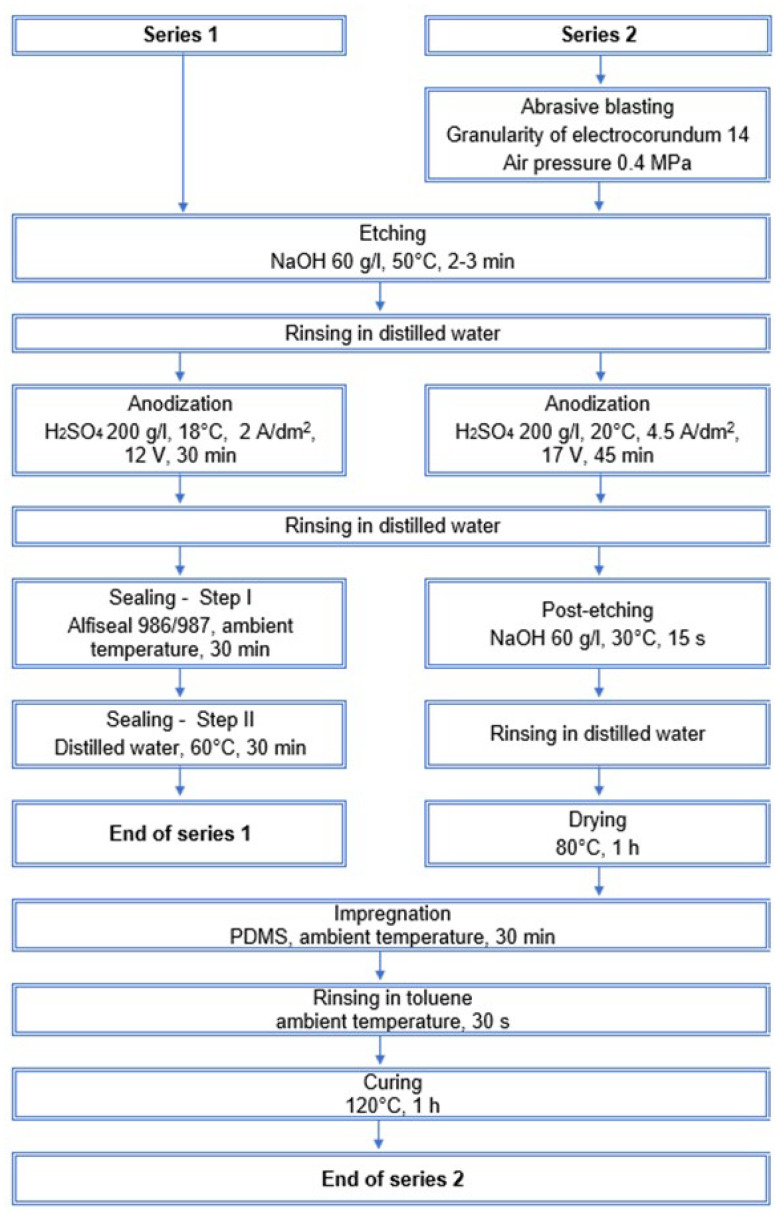
Manufacturing process.

Specimens with dimensions of 50 mm × 50 mm were produced on 1XXX series aluminum. The resulting superhydrophobic coating was compared with a coating obtained by a basic anodization process (Figure 1, Series 1).

In the case of the superhydrophobic coating, the aluminum surface was treated by abrasive blasting, anodization, impregnation by PDMS, and curing. A precise description of the manufacturing process is given in Figure 1 (Series 2). Post-etching after anodization is aimed at additional structuring of the surface. PDMS (Sylgard^®^ 184, Dow Corning, Midland, MI, USA) was used for the impregnation of the aluminum oxide coating. It was obtained by mixing a curing agent and base at a weight ratio of 1:10. Specimens from Series 2 were impregnated by immersion in PDMS for 30 min at room temperature. Immediately after removal from the container, specimens were rinsed in toluene (Chempur, Piekary Slaskie, Poland). Rinsing in toluene was aimed at the removal of PDMS excess from the surface.

Analysis of the surface was performed using scanning electron microscopy (Quanta 250 FEG SEM, FEI, Hillsboro, OR, USA). One specimen from each series (Series 1, Series 2 before impregnation, and Series 2 after impregnation) was tested using SEM. In order to improve the conductivity of the samples and enhance the SEM image quality, all specimens were plated with a 5 nm gold layer using a high-vacuum sputter (EMACE 600, Leica Microsystems, Inc, Wetzlar, Germany).

To examine the topography of the specimens’ surfaces, we used atomic force microscopy (AFM) (NT-MDT Spectrum Instruments, Moscow, Russia). One specimen from each series was tested. The measurements were conducted at ambient conditions in semi-contact mode using a silicon AFM probe (HQ:NSC15/Al BS, MikroMasch^®^ SPM Probes&Test Structures, Watsonville, CA, USA) featuring a pyramidal tip with a curvature radius of ~8 nm. The cantilever of the AFM probe was characterized by a resonance frequency range of 265–410 kHz and a force constant range of 20–80 N/m. The images were captured with a scan size of 10 µm × 10 µm and with a resolution of 256 points per line. The profile analysis and roughness calculations were carried out using Gwyddion 2.53 software.

The contact angle was measured with an optical microscope (6000 VHX, Keyence Corporation, Osaka, Japan). The volume of water drops was 3 µL during the contact angle measurements. Thickness was measured using a thickness gauge (A456 CFNFTS, Elcometer, Manchester, United Kingdom) designed for non-conductive coatings on a non-magnetic substrate.

The amount of PDMS in the coatings was calculated by weighing five specimens before anodization, after anodization, and after impregnation (CPA 225D-0CE analytical balance, Sartorius Weighing Technology, Gottingen, Germany).

**Figure 2:**
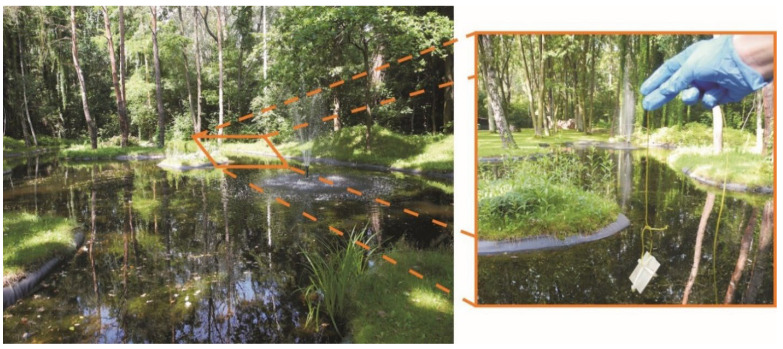
A test of antifouling properties in the pond.

The specimens were tested in terms of durability in natural conditions. One group of specimens was placed on the atmospheric corrosion test rack located at the Air Force Institute of Technology. Only the side of the specimens exposed to external factors was tested in terms of contact angle and surface tension changes. The second group of specimens was immersed in the pond located at the Air Force Institute of Technology in order to test anti-fouling properties (Figure 2). Specimens were removed periodically from the pond and air-dried. Next, photographs were taken to document the fouling of the specimens. After that, the contact angle and surface tension of the specimens were measured. The same side of the specimens was always measured and documented by means of photography. Both tests were carried out in the period from 21 July to 24 November 2021. The contact angle and surface tension were measured every seven days during both tests. However, with two exceptions, measurements were taken after about 20 days.

The surface tension of the coatings was measured using a SmartDrop-F ink set (AcXys Technologies, Saint-Martin-le-Vinoux, France). The test consisted of using the surface tension of the liquid to determine the surface tension of the material. SmartDrop-F consists of inks with a measuring range of 28–64 mN/m, developed according to the ISO 8296 standard. Surface tension measurements are commonly used to evaluate the purity of the surface.

An icing delay test was carried out using a measuring set consisting of a Peltier plate (AST-TE C40-33-006 Advanced Thermal Solutions, Norwood, Ma, USA), heatsink fan, optical microscope Keyence, and power supply (EA-El 9000T EA-Elektro-Automatik GmbH, Viersen, Germany). The specimens were placed on a cooling plate. Drops of water with a volume of 20 µL were placed on the surface of the specimen, and then the specimens were cooled from ambient temperature to −10 °C for 136 s ± 12 s. The icing delay time was measured from the start of the cooling process to the formation of characteristic peaks on the top of a water drop.

## 3. Results

**Figure 3:**
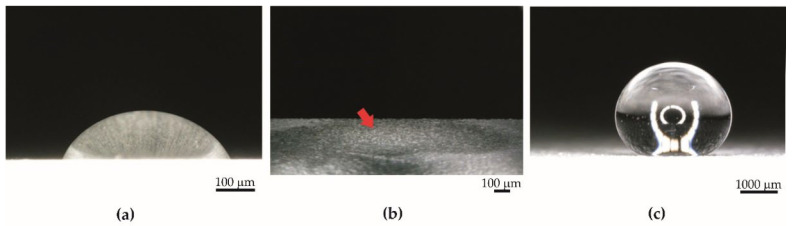
A drop of water on (**a**) hydrophilic surface (specimen from Series 1), (**b**) superhydrophilic surface (specimen from Series 2 before impregnation), (**c**) superhydrophobic surface (specimen from Series 2 after impregnation).

Specimens from Series 1 obtained by the basic anodization process showed hydrophilic properties (Figure 3a). The average contact angle for these specimens was equal to 40.59° ± 10.84°. The thickness of the coatings of Series 1 was 16.31 µm ± 0.83 µm. Typically, the untreated aluminum substrate without contamination is also hydrophilic by nature [46,47,48].

Specimens from Series 2 were obtained by impregnation of anodized aluminum oxide using PDMS. The coatings before impregnation were superhydrophilic—water was completely adsorbed on the surface (Figure 3b). However, coatings after impregnation with PDMS had superhydrophobic properties (Figure 3c), and the average contact angle was equal to 159.15° ± 2.51°. The appearance of the specimens from Series 2 was the same before and after impregnation. The PDMS layer on the surface is invisible to the naked eye. The thickness of the coatings from Series 2 was 44.94 µm ± 5.79 µm. The amount of PDMS in the coatings after impregnation, calculated by weighing specimens, was 0.22 g ± 0.10 g per 1 g of oxide coating obtained during anodization.

**Figure 4:**
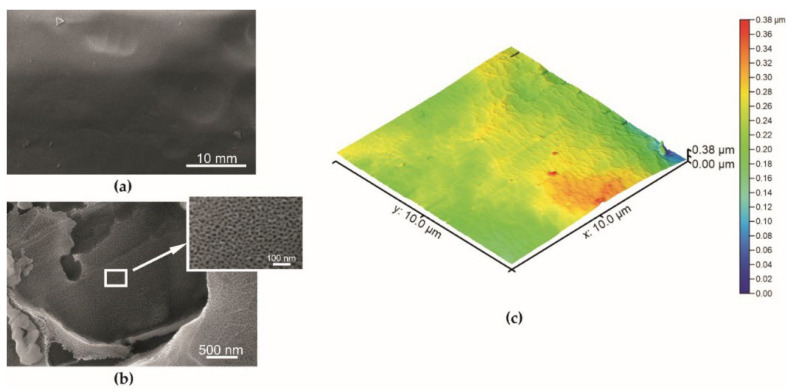
The surface of the specimen from Series 1 (basic anodization process): (**a**) mag. 10,000×: (**b**) mag. 100,000×, (**c**) AFM topography.

**Figure 5:**
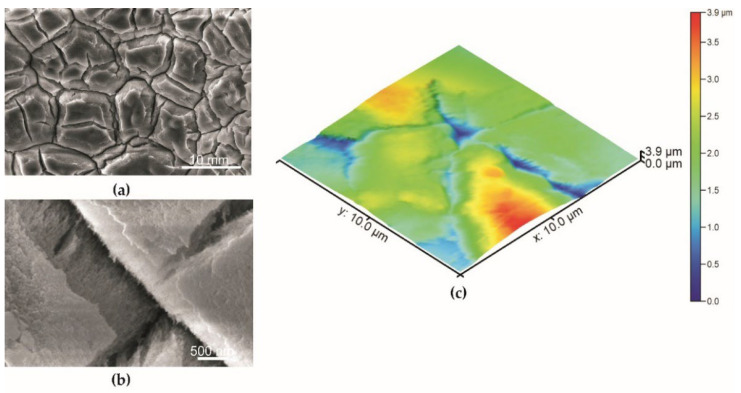
The surface of the specimen from Series 2 before impregnation by PDMS: (**a**) mag. 10,000×; (**b**) mag. 100,000×; (**c**) AFM topography.

**Figure 6:**
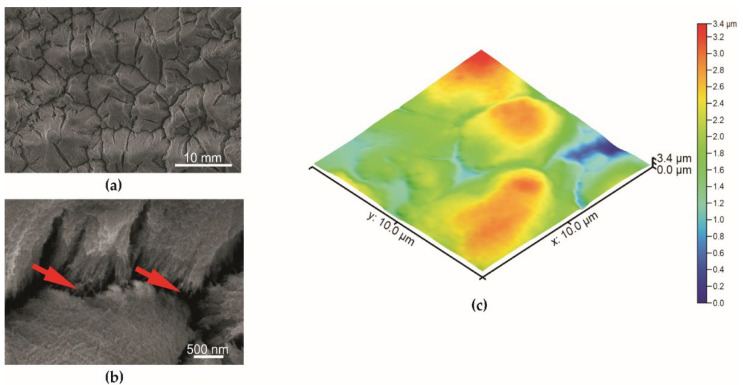
The surface of the specimen from Series 2 after impregnation by PDMS: (**a**) mag. 10,000×; (**b**) mag. 100,000×; (**c**) AFM topography.

The surface morphology of the specimens described by scanning electron microscopy is given in Figure 4, Figure 5 and Figure 6. The coating obtained during the basic anodization process is presented for comparison in Figure 4. The aluminum oxide coating is characterized by a typical nanoporous structure visible only at very high magnification (Figure 4b).

The nanoporous structure of the oxide layer with sharp edges formed on the uneven tops of the surface with a dimension from 2 µm to 10 µm on a specimen after abrasive blasting was observed. The V-shaped cavities occur between uneven tops of the structure (Figure 5a,b). In the case of a specimen with PDMS, nanoporous structures on the uneven structure with cracks are also observed (Figure 6a,b). However, the connection of nanopores of anodized aluminum oxide with PDMS causes a change in surface image. Furthermore, the polymer fibers are observed in the layer cavities of the specimen with PDMS (red arrows, Figure 6b).

**Figure 7:**
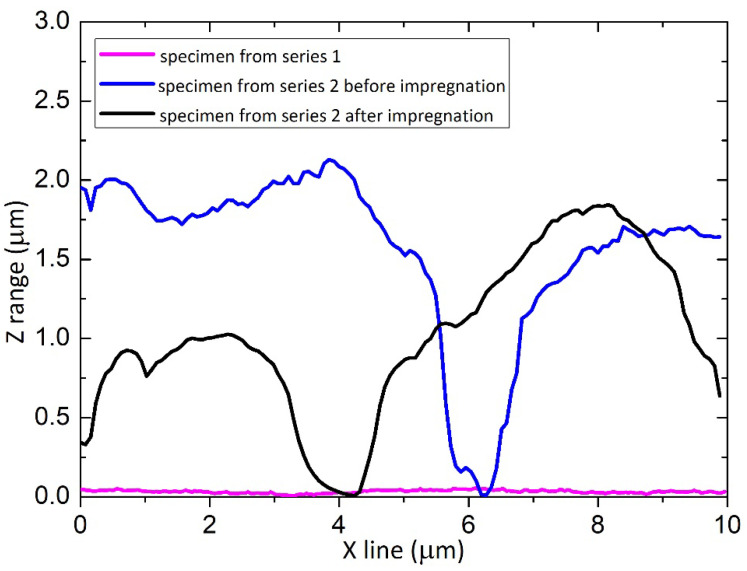
Profiles obtained from AFM topographies along the selected line for the specimen from Series 1, the specimen from Series 2 before impregnation, and the specimen from Series 2 after impregnation.

SEM and AFM techniques (Figure 4, Figure 5 and Figure 6) confirm a higher roughness of specimens from Series 2. The average roughness of specimens from Series 2 before and after impregnation is similar (Table 1). Profiles obtained from AFM topographies indicate that the coating unevenness of specimens from Series 1 does not exceed a height of 60 nm. In the case of specimens from Series 2 before and after impregnation, the unevenness of the coatings oscillates in a range of about 2 µm (Figure 7).

The climate parameters in Warsaw during the durability test in natural conditions and the anti-fouling test are given in Figure S3 in the Supplementary Materials. Average temperature, relative humidity, and precipitation were evaluated based on weather data from the website Meteoblue [49].

**Figure 8:**
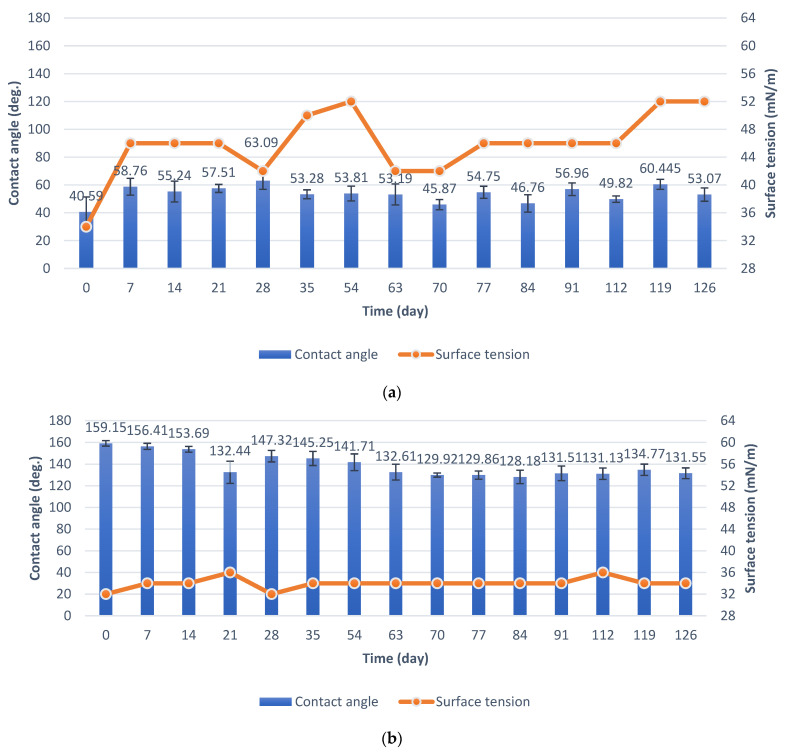
Contact angle and surface tension in the test under natural conditions: (**a**) specimens from Series 1; (**b**) specimens from Series 2.

The specimens in the durability test in natural conditions were placed on an atmospheric corrosion test rack. In the case of the basic anodization process (Series 1), the contact angle measured straight after being obtained was 40.59°. It subsequently increased to 58.76° and remained in the range of 50–60° for most of the test duration. The initial value of surface tension was 34 mN/m. However, during the test, it remained in a range of 42 to 52 mN/m (Figure 8a).

In the case of anodized aluminum modified with PDMS (Figure 8b, Series 2), the initial contact angle value was 159.15°. Values of the contact angle are related to the environmental data. A rapid reduction in contact angle was observed on the 21st day of the test, caused by a high value of precipitation and humidity during the week before the measurement (Figure S3 in Supplementary Materials). Then, the contact angle increased to 140–147° and decreased again to about 130° after 63 days, and this value remained for most of the test duration. In the case of specimens with PDMS, the initial surface tension was 32 mN/m. After that, the surface tension increased to 34 mN/m and this value remained for most of the test duration, which proves the purity of the surface, despite the decrease in the contact angle.

**Figure 9:**
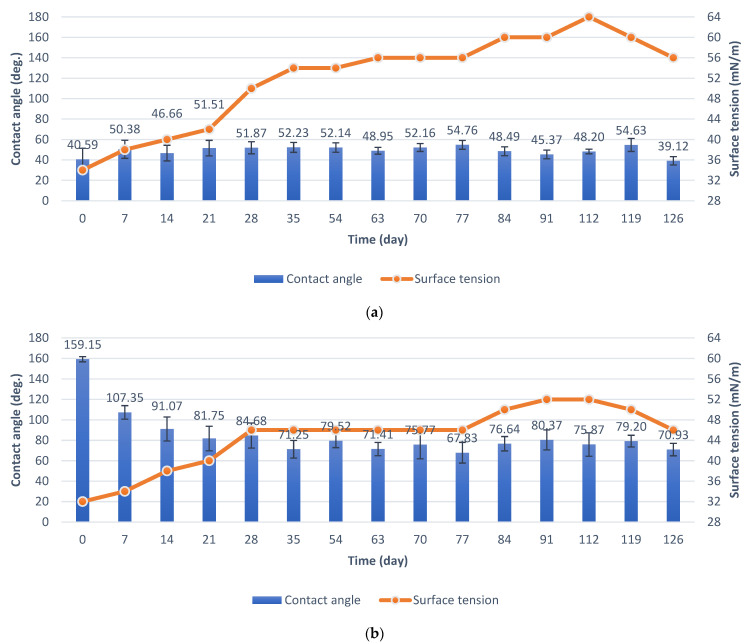
Contact angle and surface tension during the test in the pond: (**a**) specimens from Series 1, (**b**) specimens from Series 2.

Specimens were immersed in the pond in order to test their anti-fouling properties. After removing the specimens from the pond, they were air-dried, and the measurements were taken (Figure 9). In the case of aluminum specimens obtained by the basic process (Series 1), the contact angle increased from an initial value of about 40° to about 50° and remained in this range for most of the test duration. The final contact angle was 39.12°. The surface tension systematically increased from 34 mN/m to 64 mN/m, and after 119 days, the surface tension decreased, which is related to less fouling of the specimen at the end of the test (Figure 9a). The final surface tension value was 56 mN/m.

In the case of anodized aluminum specimens modified with PDMS (Series 2), the contact angle decreased from an initial value of about 159° to about 80° after 21 days of the test in the pond and remained in a range of 70° to 80° for most of the test duration. Surface tension increased from an initial value of 32 mN/m to a final value of 46 mN/m. However, the maximum surface tension value during the test was 52 mN/m. Surface tension changes are related to fouling of specimens (Figure 9b), as in the case of specimens from Series 1.

**Figure 10:**
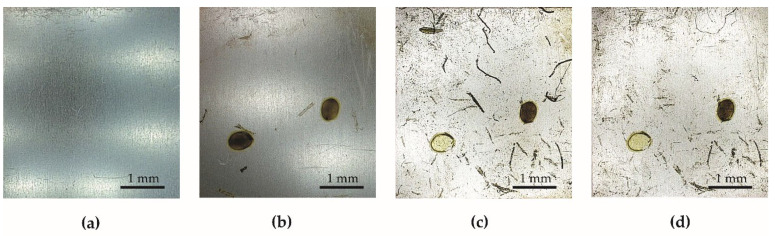
The specimen from Series 1 during the test in the pond: (**a**) before the test, (**b**) after 14 days, (**c**) after 112 days, (**d**) after 126 days (end of test).

**Figure 11:**
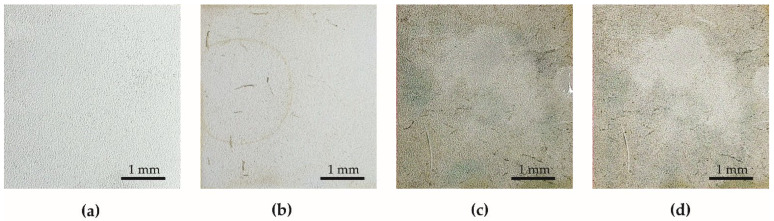
The specimen from Series 2 during the test in the pond: (**a**) before the test, (**b**) after 14 days, (**c**) after 112 days, (**d**) after 126 days (end of test).

Effects of the anti-fouling test are given in Figure 10 and Figure 11. The first fouling symptoms for both specimen series were noticed after 14 days of the test. In the case of specimens from Series 1, the fouling process is more intensive than that of the fouling of specimens from Series 2. In the case of the specimens with PDMS, only the dirt which accumulates in the rough structure of the surface is observed. The change in surface tension value (Figure 9) is related to the fouling process of the specimens given in Figure 10 and Figure 11. The surface tension increases with the fouling of the specimens. However, after 119 days of the anti-fouling test, the surface tension of both specimen series decreased. Photographs of the specimens after removal from the pond confirm a rapid decrease in the fouling (Figure 10d and Figure 11d). This effect was caused by decrease in temperature in November (Figure S3 in Supplementary Materials) and therefore less flora activity in the pond.

**Figure 12:**
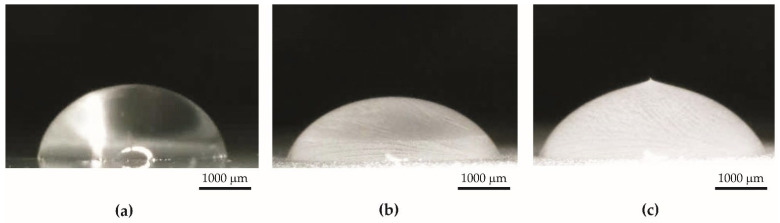
The specimen from Series 1. A drop of water on the specimen surface: (**a**) before icing, (**b**) during icing, (**c**) after complete freezing.

**Figure 13:**
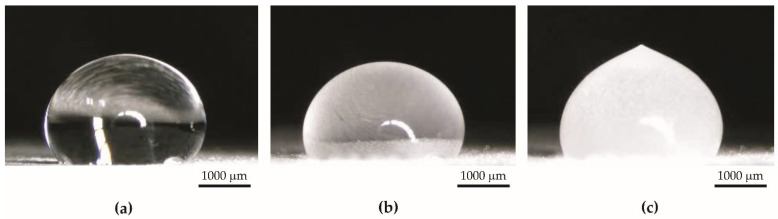
The specimen from Series 2. A drop of water on the specimen surface: (**a**) before icing, (**b**) during icing, (**c**) after complete freezing.

An icing delay test was carried out using a measuring set with a Peltier plate. The icing delay time was measured from the start of the cooling process to the forming of characteristic peaks on the top of the water drop (Figure 12 and Figure 13). The icing delay time for the specimens from Series 1 was 146 s ± 10 s, and for specimens from Series 2, it was 328 s ± 52 s.

## 4. Discussion

A superhydrophobic coating (Series 2) was obtained by the connection of three subsequent surface preparation methods—mechanical, electrochemical, and chemical. The specimens were anodized using special conditions and as a result, a superhydrophilic coating was obtained. After modification with PDMS, the superhydrophilic coating had superhydrophobic properties. The coating was obtained during an interesting process that changed the nature of the aluminum surface from hydrophobic to superhydrophilic and finally superhydrophobic. After the superhydrophobic coatings had been obtained, they had a contact angle of 159.15°. During the study, anodized aluminum modified with PDMS (Series 2) was compared with anodized aluminum obtained with the basic process (Series 1). Additionally, the superhydrophobic coating presented (Series 2) was compared with other superhydrophobic structures based on PDMS already reported in the literature. The contact angle of the superhydrophobic coating from Series 2 is similar to the contact angle of the surface presented in the study of W. Sun et al. [33] because the polydimethylsiloxane-derived film on the aluminum substrate had a contact angle of 158.7°. In another study, hierarchically structured aluminum with PDMS had a contact angle of 152° [34].

Scanning electron microscopy indicates differences in the surface structure of the specimens presented. Abrasive blasting, anodization in special conditions, and impregnation caused the forming of a structured surface of the specimens from Series 2. The average surface roughness obtained using the AFM technique for the specimen from Series 2 was 474 nm. The profile obtained from AFM topographies indicates the unevenness of the specimen from Series 2 oscillates in a range of about 2 µm. In the study of N. Atthi et al. [28], the hierarchical structures on PDMS with superhydrophobic properties are presented. The average surface roughness for the three kinds of superhydrophobic specimens was 298.9, 662.3, and 731.1 nm [28]. In the study conducted by M. Straton et al. [50], profiles obtained from AFM topographies indicate that the unevenness of superhydrophobic structures on PDMS was to be found in a range of 2–6 µm. The results of the specimens from Series 2 are analogous to the results found in the literature, despite the different methods of manufacturing superhydrophobic surfaces based on PDMS.

The durability of the superhydrophobic effect under natural conditions is usually not tested. In this study, the specimens were tested in terms of durability under natural conditions for 126 days. Specimens during the test were placed on an atmospheric corrosion test rack. The contact angle and surface tension were measured periodically during the test, which was not found to be investigated in the available literature. In the case of anodized aluminum specimens modified with PDMS (Series 2), the contact angle decreased from about 159° to about 130° after 63 days and did not change for most of the test duration. In the study of N. Atthi et al. [28], three kinds of hierarchical structures on PDMS with superhydrophobic properties were exposed to natural conditions for 7 days. After the end of the test, the contact angle values of the specimens decreased to 152.1°, 158.1°, and 156.7° [28]. The results cited [28] are analogous to the results presented in this study. After 7 days of exposure to natural conditions, the contact angle of the superhydrophobic specimens from Series 2 decreased to 156.41° (Figure 8b). However, in this study, the durability test was continued and the changes in the contact angle were already significant.

The durability of the superhydrophobic surface is not usually tested under natural conditions, but during the sandpaper abrasion test, the sand erosion test, the adhesive tape peeling test, or the scratch test [23,30,34]. In one study [34], the durability of hierarchically structured aluminum with PDMS was tested in a shear abrasion test with sandpaper, and the contact angle decreased from 152° to 143° [34].

Values of surface tension were used to evaluate the purity of the surface. The initial surface tension in the durability test under natural conditions was 32 mN/m. After that, the surface tension increased to 34 mN/m and did not change for most of the test duration, which proves the purity of the surface and its self-cleaning properties despite the decrease in the contact angle. In the available literature, the self-cleaning properties are usually evaluated during the test, which consists of contaminating the superhydrophobic specimens with dust and collecting dirt by free-sliding drops of water [33,51,52]. An evaluation of the self-cleaning properties by the periodic measurements of the specimens’ surface tension during a test under natural conditions is not found in the available literature.

The second group of specimens was immersed in the pond for 126 days in order to test anti-fouling properties. During the test, in addition to taking photographs, the contact angle and surface tension were also measured periodically. In the case of anodized aluminum specimens modified with PDMS (Series 2), the contact angle decreased from about 159° to about 80° after 21 days of the test in the pond and remained in a range of 70° to 80° for most of the test duration. The significant changes found in the contact angle of the initially superhydrophobic coating may be caused by leaching. PDMS may be removed from the coating by water during immersion in the pond.

During the anti-fouling test, the surface tension for these specimens fell in a range of 32 mN/m to 52 mN/m. Surface tension measurements were used to evaluate the fouling of the surface, which is not found in the available literature. The lower surface tension values of specimens from Series 2 and the photographic evidence confirm a cleaner surface, and thus better anti-fouling properties of specimens modified by PDMS. It is a promising result because as the literature indicates, PDMS surface without any modification shows extensive fouling after 45 days of immersion in the coastal waters [31].

Additionally, anodized aluminum specimens modified with PDMS (Series 2) had better results during the icing delay test than anodized aluminum specimens obtained in the basic process. The icing delay time of specimens from Series 2 was 328 s. However, the icing delay time of specimens with PDMS was lower than the times presented in the literature. Various types of coatings based on PMDS are characterized by icing delay times of 298 s at −10 °C [38], 600 s at −10 °C [39], 1472 s at −15 °C [40], 1800 s at −5 °C [41], 1380 s at −10 °C [41], 210 s at −15 °C [41], and 673 s from room temperature to −10 °C [42].

## 5. Conclusions

As a result of our comparative studies, the following findings are presented.

### 5.1. Durability Test in Natural Conditions

- The contact angle of aluminum anodized by the basic process increased from 40.59° to 53.07°. Surface tension increased from 34 mN/m to 52 mN/m.
- The contact angle of the superhydrophobic coating with PDMS decreased from 159.15° to 131.55°. A significant reduction in the contact angle occurred after 63 days of the test. Surface tension increased from 32 mN/m to 34 mN/m. The coating had sufficient self-cleaning properties, despite the loss of superhydrophobicity.

### 5.2. Anti-Fouling Test in the Pond

- The contact angle of aluminum anodized by the basic process decreased from 40.59° to 39.12°. Surface tension increased from 34 mN/m to 56 mN/m.
- The contact angle of superhydrophobic coating with PDMS decreased from 159.15° to 70.93°. Surface tension increased from 32 mN/m to 44 mN/m. The specimens with PDMS had better anti-fouling properties.

### 5.3. Anti-Icing Test

- Icing delay time for aluminum anodized by the basic process was 146 s ± 10 s. For the superhydrophobic coating with PDMS, the icing delay time was 328 s ± 52 s.

Based on the results, it is concluded that superhydrophobic coating with PDMS has potential anti-fouling and icing delay applications. However, increased durability of the superhydrophobic effect is required.

## Figures and Tables

**Table 1:** Average surface roughness value of specimens.

Specimen	S_a_—Average Surface Roughness (nm)
Series 1	25.5 ± 3.5
Series 2 before impregnation	484.5 ± 42.4
Series 2 after impregnation	474.0 ± 38.8

## Data Availability

The data presented in this study are available upon request from the corresponding author.

## Supplementary Materials

The following supporting information can be downloaded at: https://www.mdpi.com/article/10.3390/ma15031042/s1. The theoretical background concerning wettability and the contact angle, and the climate parameters in Warsaw during the durability test in natural conditions and the anti-fouling test can be found in the Supplementary Materials. Figure S1: Wettability of surface (a) superhydrophilic surface θ < 10°, (b) hydrophilic surface θ < 90°, (c) hydrophobic surface θ > 90°, (d) superhydrophobic surface θ > 150°; Figure S2: A drop of water on (a) flat surface—Young model, (b) rough surface—Wenzel model, (c) nano/micro-structured surface—Cassie-Baxter model, (d) nano/micro-structured surface—Metastable model; Figure S3: Average temperature, relative humidity, and precipitation in Warsaw in the period from 21 July to 24 November 2021. References [53,54,55,56,57,58,59] are cited in the Supplementary Materials.
