# Supplementary material for: Superhydrophobic Coating Based on Porous Aluminum Oxide Modified by Polydimethylsiloxane (PDMS)
Source: Materials (Basel). 2022 Jan 28;15(3):1042. doi: 10.3390/ma15031042 (PMC8840686; doi:10.3390/ma15031042)
Supplement: Supplementary file 1 [file materials-15-01042-s001.zip › materials-1559136-supplementary.pdf]

materials

Supplementary Material

Superhydrophobic Coating Based on Porous Aluminum Oxide Modified by Polydimethylsiloxane (PDMS)

Klaudia Olkowicz [1,*], Zofia Buczko [2], Barbara Nasiłowska [3], Kamil Kowalczyk [1] and Joanna Czwartos [3]

[1] Air Force Institute of Technology, 01-494 Warsaw, Poland; kamil.kowalczyk@itwl.pl
[2] Łukasiewicz—Institute of Precision Mechanics, 01-796 Warsaw, Poland; zofia.buczko@imp.lukasiewicz.gov.pl
[3] Institute of Optoelectronics, Military University of Technology, 00-908 Warsaw, Poland; barbara.nasilowska@wat.edu.pl (B.N.); joanna.czwartos@wat.edu.pl (J.C.)
* Correspondence: klaudia.olkowicz@itwl.pl

Theoretical background of wettability

Wettability of a surface is characterized by the contact angle θ. The value of the contact angle depends on the interaction between the interfacial surface tension of the solid, vapor (air), and liquid (Figure S1). Superhydrophilic surfaces are characterized by a contact angle below 10°, hydrophilic by a contact angle below 90°, and hydrophobic by a contact angle above 90°. However, superhydrophobic surfaces have a contact angle above 150° and are completely water-repellent [1-3].

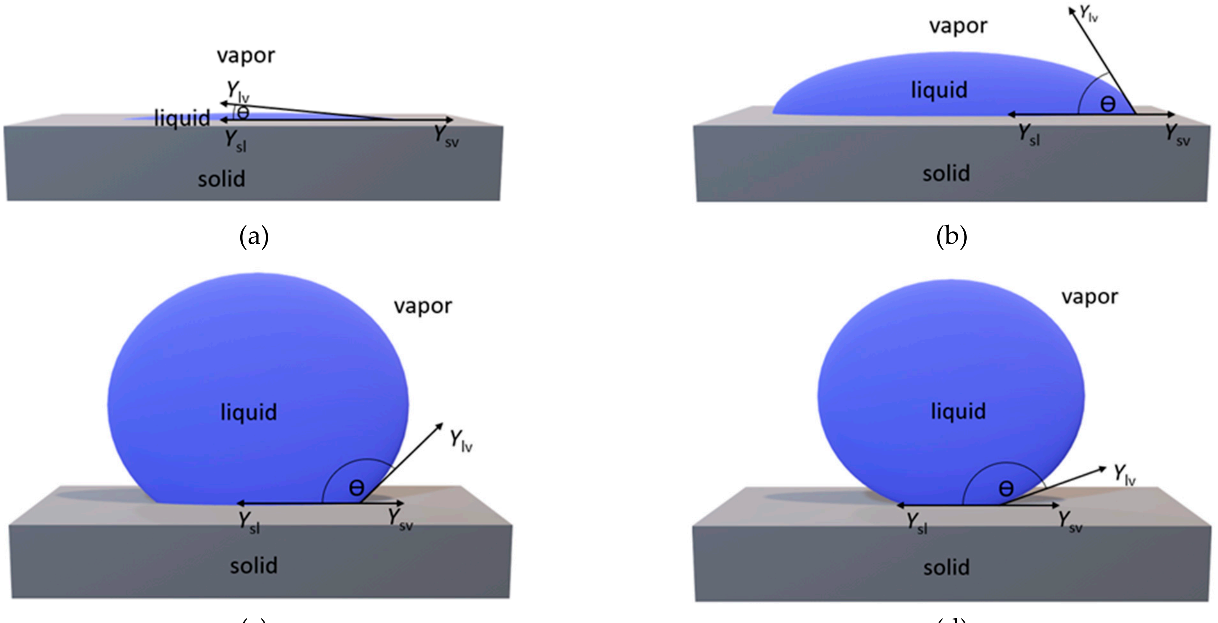

Figure S1. Wettability of surface (**a**) superhydrophilic surface θ<10°, (**b**) hydrophilic surface θ<90°, (**c**) hydrophobic surface θ>90°, (**d**) superhydrophobic surface θ>150°.

Interaction between contact angle and interfacial surface tensions is given by Young equation (S1) [4-5]:

$$\cos\theta_Y = \frac{\gamma_{sv} - \gamma_{sl}}{\gamma_{lv}} \quad \text{(S1)}$$

where: θ_Y – contact angle in Young model; Υ_{sv} - interfacial surface tension of solid-vapor; Υ_{sl} - interfacial surface tension of solid-liquid; and Υ_{lv} - interfacial surface tension of liquid vapor.

The Young equation applies to flat surfaces of a solid state (Figure S2a) [5]. In the case of rough or porous substrates, the value of the contact angle may change, because the contact angle depends on surface formation. The wettability of a rough surface is given by the Wenzel equation (S2). The Wenzel equation includes the impact of surface formation on strengthening effect of hydrophilicity and hydrophobicity (Figure S2b) [2,5-7]:

$$cos\theta_w = r\frac{\gamma_{sv}-\gamma_{sl}}{\gamma_{lv}} = rcos\theta_Y \quad (S2)$$

where: θ_W – contact angle for a structured surface in Wenzel model; θ_Y – contact angle for a flat surface in Young model; and r – roughness ratio.

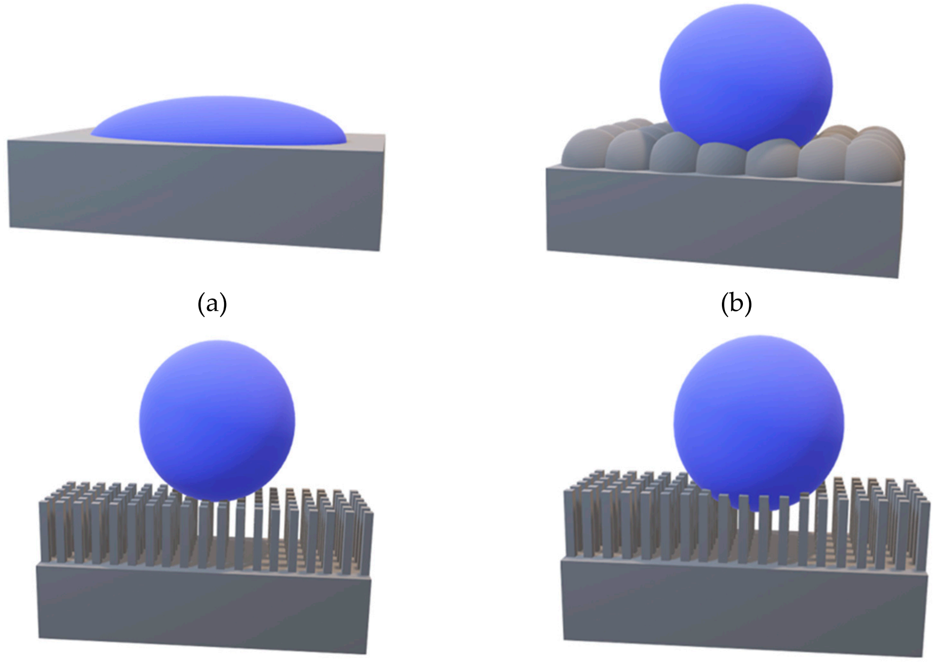

(a) (b)

(c) (d)

Figure S2. A drop of water on (**a**) flat surface – Young model, (**b**) rough surface – Wenzel model, (**c**) nano/micro-structured surface – Cassie-Baxter model, (**d**) nano/micro-structured surface – Metastable model.

In the Cassie-Baxter model, a drop of water is placed on top of a structured substrate and does not penetrate the nano and micro cavities of the structure. Therefore, it does not wet the entire structure (Figure S2c). The contact angle in the Cassie-Baxter model depends on the interaction between liquid, solid, and air in the nano/miro cavities of the structure, and is given by equation (S3) [2,5,7,8]:

$$cos\theta_{CB} = fcos\theta_1 + (1-f)cos\theta_2 \quad (S3)$$

where: θ_{CB} – contact angle in Cassie-Baxter model; f - fraction of the surface area of the substrate which is wetted by liquid; θ_1 – contact angle of substrate; and θ_2 – contact angle of substance in the nano/mirco substrate.

If the substance in the nano/micro cavities of the structure is air, then θ_2 is equal to 180° and the Cassie-Baxter equation is given by (S4) [2, 5]:

$$cos\theta_{CB} = f(cos\theta_1 + 1) - 1 \quad (S4)$$

However, the Cassie-Baxter model is rarely applied, because frequently nano and mirco cavities in the structure are partly wetted by water (Figure S2d). The Metastable model was discussed in the literature and is given by equation (S5) [9-12]:

$$cos\theta_M = rfcos\theta_1 + (1-f)cos\theta_2 \quad (S5)$$

where: θ_M – contact angle in the Metastable model.

Theoretical background of wettability

The climate parameters in Warsaw during the durability test in natural conditions and the anti-fouling tests are given in Figure S3. Average temperature, relative humidity, and precipitation were evaluated based on weather data from the website Meteoblue [13].

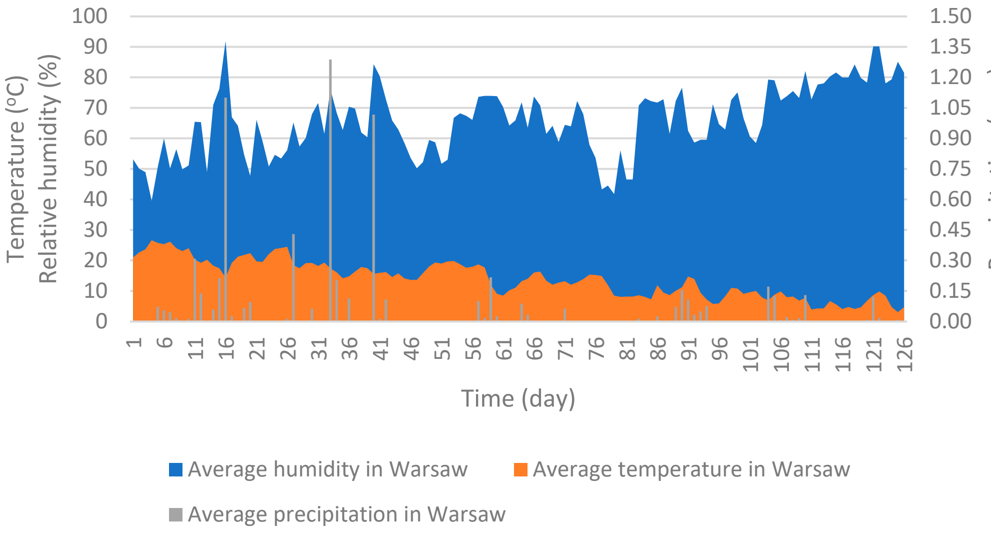

Figure S3. Average temperature, relative humidity, and precipitation in Warsaw in the period from 21 July to 24 November 2021.

References

1. Roach, P., Shirtcliffe, N.J., Newton, M.I. Progress in superhydrophobic surface development. *Soft Mater* **2008**, 4, pp. 224-240.
2. Buczko, Z., Olkowicz, K., Tomassi, P., Zolciak, T. Coatings with carbon nanostructures with hydrophobic properties made by anodizing and the CVD method on stainless steel. *Inzynieria Powierzchni* **2018**, 23, pp. 30-37.
3. Alam, K., Khan, K.I, Ullah, A., Ullah, A., Ali, S., Ullah, S., Ali, A., Hussain, S. Fabrication of superhydrophillic and graded index antireflective double layer coating for solar photovoltaics module using aerosol impact deposition assembly. *Thin Solid Films* **2021**, 721, pp. 138518.
4. Young, T**.** III. An essay on the cohesion of fluids. *Philosophical Transactions of the Royal Society of London* **1805**, 95, pp. 65-87.
5. Li, W., Zhan, Y., Yu, S. Applications of superhydrophobic coatings in anti-icing: Theory, mechanisms, impact factors, challenges and perspectives. *Progress in Organic Coatings* **2021**, 152, pp. 106117.
6. Wenzel, R.N. Resistance of solid surfaces to wetting by water. *Industrial and Engineering Chemistry* **1936**, 28, pp. 988-994.
7. Atthi, N., Sripumkhai, W., Pattamang, P., Thongsook, O., Meananeatra, R., Saengdee, P., Srihapat, A., Supadech, J., Janseng, T., Maneesong, A., Kongto, N., Rodchanarowan, A., Klunngien, N., Jeamsaksiri, W**.** Superhydrophobic and superoleophobic properties enhancement on PDMS micro-structure using simple flame treatment method. *Microeletronic Engineering* **2020**, 230, pp. 111362.
8. Cassie, A.B.D., Baxter, S. Wettability of porous surfaces. *Transaction of the Faraday Society* **1944**, 40, pp. 546-551.
9. Miwa, M., Nakajima, A., Fujishima, A., Hashimoto, K., Watanabe, T. Effects of the surface roughness on sliding angles of water droplets on superhydrophobic surfaces. *Langmuir* **2000**, 16, pp. 5754-5760.
10. Marmur, A. Wetting on hydrophobic rough surfaces: to be heterogeneous or not to be? *Langmuir* **2003**, 19, pp. 8343-8348.
11. Buczko, Z., Olkowicz, K., Krasucki, J., Grabowiecki, K., Osuchowska, E., Tomassi, P. Superhydrophobic properties of aluminium produced by surface abrasive blasting, anodic oxidation and fatty acid impregnation. *Transactions of the IMF* **2021**, 99, pp. 73-79.
12. Yong, J., Yang, Q., Chen, F., Zhang, D., Du, G., Bian, H., Si, J., Yun, F., Hou, X**.** Superhydrophobic PDMS surfaces with three-dimensional (3D) pattern-dependent controllable adhesion. *Applied Surface Science* **2014**, 288, pp. 579-583.
13. Meteoblue. Available online: https://www.meteoblue.com/ (accessed on 25 November 2021).
